# Syphilis Infection Differentially Regulates the Phenotype and Function of γδ T Cells in HIV-1-Infected Patients Depends on the HIV-1 Disease Stage

**DOI:** 10.3389/fimmu.2017.00991

**Published:** 2017-08-21

**Authors:** Zhen Li, Xiaofan Lu, Zhiliang Hu, Zhenwu Luo, Wei Jiang, Hao Wu, Yanqing Gao, Junling Yan, Qiuyue Zhang, Aixin Song, Xiaojie Huang, Danlei Mou, Bin Su, Tong Zhang

**Affiliations:** ^1^Beijing Key Laboratory for HIV/AIDS Research, Center for Infectious Diseases, Beijing You’an Hospital, Capital Medical University, Beijing, China; ^2^Department of Infectious Diseases, The Second Affiliated Hospital, Southeast University, Nanjing, China; ^3^Department of Microbiology and Immunology, Medical University of South Carolina, Charleston, SC, United States; ^4^Division of Infectious Diseases, Department of Medicine, Medical University of South Carolina, Charleston, SC, United States; ^5^Department of Dermatology, Center for Infectious Diseases, Beijing You’an Hospital, Capital Medical University, Beijing, China

**Keywords:** syphilis, acute/chronic HIV-1 infection, γδ T cells, innate immune response, IL-17

## Abstract

A rapidly escalating outbreak of syphilis infection has been affected men who have sex with men, particularly those with HIV-1 infection. γδ T cells are unconventional immune cells with two main subsets, Vδ1 T cells and Vδ2 T cells, which possess a combination of innate and adaptive immune features allowing them against HIV-1. However, whether syphilis infection affects the phenotype and function of γδ T cells in HIV-1-infected patients remains unclear, especially in acute HIV-1 infection (AHI). In this study, we enrolled 57 HIV-1-infected patients (24 with HIV-1 infection only and 33 coinfected with syphilis) from an acute HIV-1-infected cohort in Beijing (PRIMO). A comprehensive analysis of γδ T-cell phenotype and function was performed by flow cytometry. We found syphilis coinfection could reverse the imbalance of Vδ1/Vδ2 ratio in AHI. Syphilis infection results in decreased γδ T-cell activation in AHI, but increased γδ T-cell activation in chronic HIV-1 infection (CHI). Moreover, patients with CHI had larger numbers of IL-17-producing γδ T cells than those with AHI, regardless of syphilis status. Thus, syphilis affected the γδ T-cell immune response differently in patients depending on the stages of HIV-1 disease. In addition, the percentage of IL-17-producing γδ T cells was positively correlated with the percentage of neutrophils. These results suggest that the γδ T-cell/IL-17/neutrophil axis is involved in HIV-1 pathogenesis and disease progression. Taken together, our observations provide new insight into the roles of γδ T cells in immunopathogenesis of syphilis and HIV-1 coinfection, particularly during AHI, and our findings may be helpful for the prevention of syphilis and other sexually transmitted infections and highlight the great significance on the remedy of patients coinfected with HIV-1.

## Introduction

Syphilis is a chronic bacterial infection caused by the sexually transmitted pathogen, *Treponema pallidum*. The incidence of syphilis has increased sharply in recent years in men who have sex with men (MSM), especially those with HIV-1 infection (1–7). Syphilis worsens disease progression in HIV-1-infected patients, as demonstrated by CD4^+^ T-cell depletion and increased plasma HIV-1 RNA levels (8–10). Syphilis may also increase the risk of HIV-1 infection and transmission in MSM population (11, 12). Meanwhile, HIV-1 infection may effect on the presentation, disease progression, and efficacy of treatment on syphilis (13). The host immune system plays a key role in controlling HIV-1 disease progression through host restriction factors (TRIMs, APOBEC3G, SAMHD1), cytokines (IL-6, IFN-γ, TNF-α), and chemokines (MIP-1α, MIP-1β, RANTES) (14–18). Cells producing IL-17 and IFN-γ have been implicated in inflammation and central nervous system damages (19, 20). However, the cellular immune responses elicited by HIV-1 and syphilis coinfection have not been well studied.

γδ T cells account for only a small proportion (1–10%) of T lymphocytes, but are nevertheless a critical component of the innate immune system and play important roles in the first line (21). Human γδ T cells have been defined into two main subsets, Vδ_1_ T cells and Vδ_2_ T cells. Vδ_1_ γδ T cells are located predominantly in the mucosae, where they recognize stress-induced molecules (such as ULBPs and MICA/B), and play a protective role. By contrast, Vδ_2_ γδ T cells recognize phosphoantigens (such as IPP). They account for most of the circulating γδ T cells in the bloodstream and are directed against tumors and infectious diseases. In the peripheral blood, Vδ_1_ T cells, also known as regulatory γδ T cells, play an immunosuppressive role, through Foxp3 expression or the secretion of cytokines, such as TGF-β and IL-10 (22). On the contrary, Vδ_2_ T cells mainly served as a control of antiviral immunity; cytotoxic cells kill tumor cells or pathogen-infected cells by secreting cytokines, perforin, granzyme B, or through Fas-FasL pathway; antigen presentation; or B helper T-cell function (21, 23, 24). HIV-1 infection strongly depletes the numbers of Vδ_2_ T cells and induces Vδ_2_ T-cell anergy, resulting in weaker responses to IPP stimulation (25). In our previous study, we found that γδ T cells were over-activated in HIV-1 infection and were associated with HIV-1 disease progression (26). IL-17-producing γδ T cells increased in HIV-1-infected patients with fast disease progression and were involved in HIV-1 pathogenesis (26). However, to our knowledge, there are few studies describing the antisyphilitic role of γδ T cells in HIV-1 infection. In addition, increasing IFN-γ/IL-17-producing CD8^+^ T cells were considered as a compensation in HIV^+^ individuals, but they were not sufficient to eliminate the spirochete (27). Therefore, we propose that γδ T cells involved in cell-mediated antisyphilitic response in HIV-1-infected patients. However, how the phenotype and function of γδ T cells in HIV-1-infected patients with syphilis coinfection change remains unclear, especially in acute HIV-1 infection (AHI).

In the current study, we performed a comprehensive analysis of γδ T-cell phenotype and function in patients coinfected with syphilis and HIV-1. We found that syphilis affected the phenotype and function of γδ T cells differently in HIV-1-infected patients, depending on the stages of HIV-1 disease. Our observations provide new insight into the roles of γδ T cells in immunopathogenesis of syphilis and HIV-1 coinfection. These findings suggest syphilis monitoring, follow-up, and treatment in MSM or HIV-1-positive individuals should be enhanced.

## Materials and Methods

### Study Subjects

**Figure 1:**
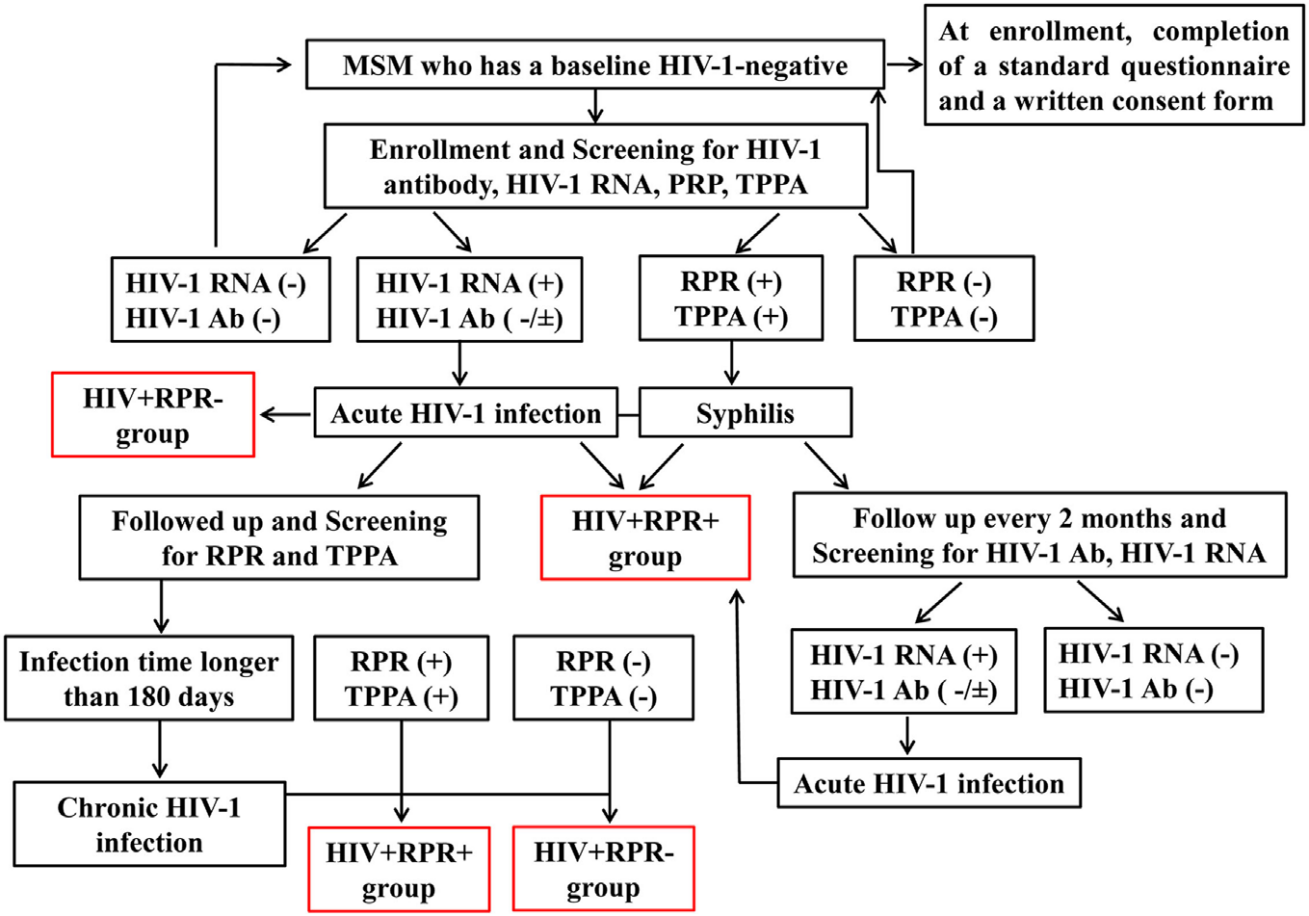
The flow chart summarizing the enrollment of subjects in the study. HIV-1-negative men who have sex with men (MSM) were enrolled in a prospective cohort study to identify acute HIV-1 infection. At enrollment, participants were screened for HIV-1 infection and syphilis by detecting HIV-1 RNA, HIV-1 antibody, rapid plasma reagin (RPR), and *T. pallidum* particle agglutination assay (TPPA) respectively. Then they were followed up every 2 months and HIV-1RNA, HIV-1 antibody, RPR, and TPPA were tested at each visit. Once acute HIV-1-infected individuals were captured, they continued to be followed up to 2–3 years to observe the natural progression of HIV-1 infection. According to the Fiebig stage, when the estimate date of infection was longer than 180 days, it is considered as a chronic HIV-1 infection. Acute or chronic HIV-1-infected patients with RPR^+^ were separated into HIV^+^RPR^+^ group in acute or chronic infection. Otherwise, they were enrolled into HIV^+^RPR^−^ group in acute or chronic infection.

Study subjects used in this study were enrolled from the Beijing PRIMO Clinical Cohort, a prospective study cohort of HIV-1-negative MSM to identify AHI at Beijing You’an Hospital, Beijing, China, since October 2006 (28). After enrollment, they were screened for plasma HIV-1 antibody, HIV-1 RNA level, clinical signs of acute infection, and syphilis status. Then they were followed up every 2 months, and HIV-1 antibody and syphilis were detected at each visit. AHI was defined as a negative or indeterminate HIV-1 antibody status but a positive HIV RNA (29). Once acute HIV-1-infected cases were identified, patients were followed up at 1, 2, 4, 8, and 12 weeks, and then every 3 months and syphilis status was monitored at each visit (Figure 1). Blood samples were collected, and peripheral blood mononuclear cells (PBMCs) and plasma were isolated and cryopreserved. Estimated date of infection was defined as the mid-point between the last HIV-1 antibody negative test and the first HIV-1 antibody positive test, or as 14 days prior to a positive RNA PCR assay on the same day as a negative HIV Enzyme Immunoassay. Patients whose infection time was longer than 180 days were defined as chronic HIV-1 infection (CHI). 26 AHI and 31 CHI patients were recruited randomly. The estimated infection date of AHI and CHI patients were 62 ± 33 and 565 ± 307 days, respectively. Early HIV-1 infection can be depicted as six discrete stages proposed by Fiebig et al. (30, 31). Stage I–II: HIV-1 RNA positive, ELISA negative with 3 patients; stage III–IV: HIV-1 RNA positive, ELISA positive, and Western blot negative or indeterminate with 4 patients; stage V–VI: HIV-1 RNA positive, ELISA positive, Western blot without/with p31 band with 19 patients. Syphilis was diagnosed on the basis of a compatible history, and the results of rapid plasma reagin (RPR) test (Shanghai Kehua Company, China) and *T. pallidum* particle agglutination assay (TPPA) (Fujirebio Diagnostics, Inc., Japan) (32). Based on the RPR and TPPA results, AHI and CHI patients were, divided into HIV^+^RPR^+^ group (*n* = 15, *n* = 18) and HIV^+^RPR^−^ group (*n* = 11, *n* = 13), respectively. All AHI and CHI patients were ART-naïve treated. 6 patients in acute HIV^+^RPR^+^ group and 4 patients in chronic HIV^+^RPR^+^ group received benzathine benzylpenicillin treatment for syphilis before their enrollment in this study. Patients with opportunistic infections or coinfections with tuberculosis, hepatitis B virus, or hepatitis C virus were excluded. Twenty age-matched male MSM HIV-1-negative controls (HC) were included as controls. The characteristics of all subjects are described in the Table 1.

**Table 1:** Basic characteristics of all participants enrolled in this study.

Characteristics	Healthy control	Acute HIV-1 infection		*P-*value	Chronic HIV-1 infection		*P-*value
		RPR^+^	RPR^−^		RPR^+^	RPR^−^	
Cases	20	15	11		18	13	
Chinese Han population (%)	100	100	100		100	100	
Age (years)	29.5 ± 7.7	31.5 ± 6.8	30.3 ± 7.5	0.678	34.6 ± 7.6	33.6 ± 8.8	0.703
Infection time (day)	NA	58.6 ± 23.8	66.6 ± 43.8	0.917	500.9 ± 249.4	655.4 ± 364.1	0.483
CD3^+^ T-cell count (cells/μl)	1751 ± 467	1622 ± 499	1545 ± 537	0.958	1801 ± 846	1629 ± 394	0.857
CD4^+^ T-cell count (cells/μl)	865 ± 300**	423 ± 150	438 ± 144	0.815	415 ± 118	521 ± 143	0.097
CD8^+^ T-cell count (cells/μl)	824 ± 291	1173 ± 503	1024 ± 396	0.716	1072 ± 558	1070 ± 397	0.561
CD4:CD8 ratio	1.13 ± 0.55**	0.38 ± 0.22	0.49 ± 0.23	0.203	0.37 ± 0.27	0.56 ± 0.32	0.063
Plasma viral load (log10 copies/ml)	NA	4.45 ± 0.74	4.66 ± 1.15	0.978	4.27 ± 0.88	3.86 ± 0.69	0.267

*Data were presented as mean ± SD*.

**P < 0.001, compared with HIV-1-infected patients.

*P values were determined by Mann–Whitney test*.

*NA, not applicable; RPR, rapid plasma reagin*.

This study and all relevant experiments have been approved by the Beijing You’an Hospital Research Ethics Committee and written informed consent was obtained from each participant according to the Declaration of Helsinki. All the participants provided written informed consent for their information, and clinical samples were stored and used for research. At enrollment, all subjects provided baseline demographic, clinical, and epidemiological information by completing a standardized questionnaire. The methods were carried out in accordance with approved guidelines and regulations.

### Whole-Blood Analysis

Venous blood was collected into tubes containing EDTA as an anticoagulant. Whole blood cells analysis was performed on Uritest-3000 Fully Automated Hematology Analyzer (URIT medical electronic company, Shenzhen, China). Absolute counts of CD3^+^, CD4^+^, and CD8^+^ T lymphocytes were obtained as previously described (26).

### Viral Load Testing

Plasma HIV-1 viral load (copies/ml) were quantified using the COBAS AmpliPrep/COBAS TaqMan 48 Analyser (Roche Diagnostic, Branchburg, NJ, USA), or by real-time PCR (Abbott, Des Plaines, IL, USA), with a detection limit of 40 copies/ml of plasma.

### Serological Assays

HIV-1 infection status was determined by screening with a HIV-1/2 antigen/antibody combo enzyme immunoassay (Beijing Wantai Biological Medical Company, Beijing, China). Positive specimens were further conformed by using Western blot for HIV-1/2 (HIV Blot 2.0 MP Diagnostics, Singapore). The diagnosis of syphilis was performed with the RPR test (Shanghai Kehua Company, China) and TPPA (Fujirebio Diagnostics, Inc., Japan) (32). A seropositive result for TPPA was defined as the presence of a past or current syphilis infection, while a seropositive result for both TPPA and RPR was diagnosed as a current syphilis infection.

### Antibodies

Phycoerythrin (PE)-conjugated anti-human CD38 (HIT2) monoclonal antibody (mAb), allophycocyanin (APC)-conjugated anti-human HLA-DR (L243) mAb, phycoerythrin-cyanine 7 (PE-cy7)-conjugated anti-human CD3 (HIT3a) mAb, peridinin-chlorophyll protein complex cyanine 5.5 (PerCP-cy5.5)-conjugated anti-human CD27 (O323) mAb, and APC-conjugated anti-human CD45RA (HI100) mAb were purchased from Biolegend (San Diego, CA, USA). Fluorescein isothiocyanate (FITC)-conjugated anti-human pan TCRγδ mAb, PE-conjugated anti-human pan TCRγδ (IMMU510) mAb and FITC-conjugated anti-human pan Vδ_2_TCR mAb (IMMU389) were purchased from Immunotech (Beckman Coulter, Fullerton, France). FITC-conjugated anti-human panVδ_1_TCR (TS8.2) mAb was purchased from Pierce (Rockford, IL, USA). PE-conjugated IL-17A (SCPL1362) mAb and APC-conjugated interferon-γ (IFN-γ) (B27) mAb were purchased from BD Pharmingen (San Diego, CA, USA). The isotype control mAbs were purchased from the corresponding companies.

### Flow Cytometry

**Figure 2:**
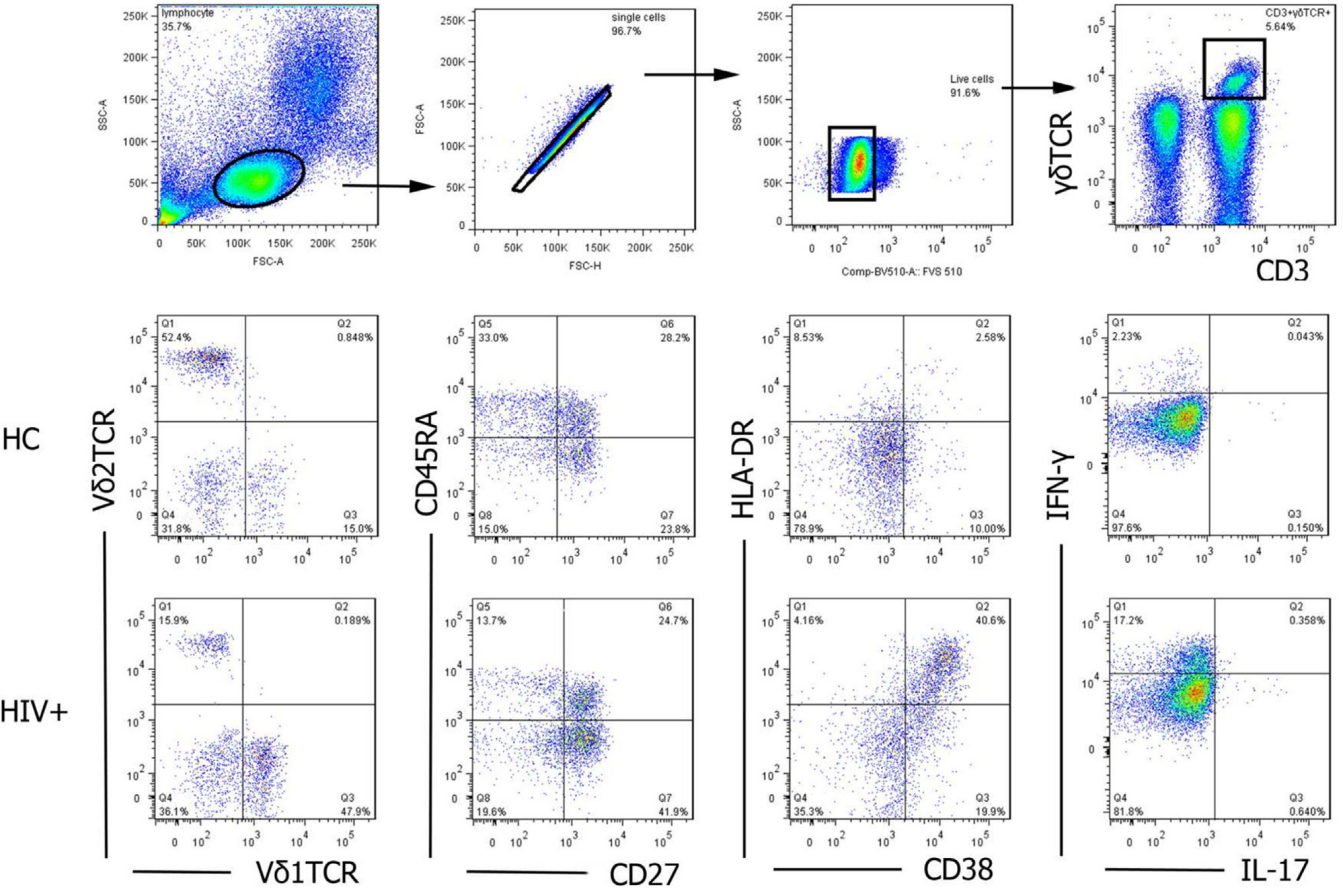
The gating strategy for flow cytometric analysis of γδ T cells. Among all events, forward angle and side scatter light gating were gated on lymphocytes and were used to exclude cell debris from the analysis. Forward height and forward area were used to exclude doublet cells, and cells were labeled with LIVE/DEAD fixable viability stain 510, and dead cells were excluded. Then, CD3^+^γδTCR^+^cells, Vδ1 and Vδ2 subsets, functional subsets (T_Naive_, T_CM_, T_EM_, and T_EMRA_), activation (CD38 and HLA-DR), and cytokines secretion (IL-17 and IFN-γ) were gated, analyzed, and compared between healthy controls (HC) and HIV-1-infected individuals. The final analysis was performed with FlowJo software, which generated a graphical output. FSC, forward scatter; SSC, side scatter.

For surface staining, PBMCs were isolated from HC and HIV-1-infected patients with or without syphilis infection. Cells were washed with 1% bovine serum albumin in PBS and were labeled with LIVE/DEAD fixable viability stain 510 (BD Biosciences, San Jose, CA, USA), and dead cells were excluded. Then, cells labeled with specific surface antibodies: gating strategy on CD3^+^γδTCR^+^ cells, Vδ_1_ and Vδ_2_ T cells, or γδT_Naive_ (CD27^+^CD45RA^+^), γδT_CM_ (CD27^+^CD45RA^−^), γδT_EM_ (CD27^−^CD45RA^−^), and γδT_EMRA_ (CD27^−^CD45RA^+^) were displayed (Figure 2). Cytometer setup and tracking calibration particles were used to ensure that fluorescence intensity measurement was consistent in all experiments. Flow cytometry Comp-Beads kits (BD Biosciences, San Jose, CA, USA) were used for compensation. Forward angle and side scatter light gating were gated on lymphocytes and were used to exclude cell debris from the analysis. Forward height and forward area were used to exclude doublet cells. Cells were performed with a FACScan flow cytometer, as previously described (33, 34). The final analysis was performed with FlowJo software (version 7.6.2; Tree Star Inc., Ashland, OR, USA), which generated a graphical output. The strategies for the analysis of flow cytometry data are detailed in Figure 2.

### Intracellular Staining

Peripheral blood mononuclear cells were incubated with 50 ng/ml phorbol 12-myristate-13-acetate (PMA), 1 μg/ml ionomycin, and 10 μg/ml brefeldinA (Sigma, St Louis, MO, USA) at 37°C for 6 h. Cells were collected, washed, and labeled with specific surface antibodies. Then, cells were washed, fixed, permeabilized, and incubated with IL-17 and IFN-γ mAbs. Cells were performed with a FACScan flow cytometer. Cells were first gated on 510 negative cells (live cells), then gated on CD3^+^γδTCR^+^ cells, the expression of IL-17 or IFN-γ was analyzed (Figure 2). Data were analyzed by FlowJo software, as described above.

### Statistical Analysis

Data are expressed as mean ± SD. Statistical analysis was performed with GraphPad Prism software version 5.03 (GraphPad Software, San Diego, CA, USA). Statistical significance *P* values for differences between groups were assessed by Mann–Whitney tests and one-way ANOVA test. The statistical dependence between variables was assessed by performing Spearman’s rank correlation analysis. All tests were two-tailed, and values of *P* < 0.05 were considered significant.

## Results

### Characteristics of Participants

26 AHI, 31 CHI patients, and 20 HIV-1-negative HC were enrolled in this study (Table 1). Both AHI and CHI patients were further divided into two groups (HIV^+^RPR^+^ or HIV^+^RPR^−^) based on the syphilis status. The information of all the subjects is presented in Table 1. The age, sex, and the nationality of the HIV-1-infected patients and HC are matched. The estimated date of infection and viral load of HC are unavailable. The absolute number of CD4^+^ T cells and CD4/CD8 ratio in AHI and CHI patients are much lower than that in HC (*P* < 0.001). The absolute number of CD3^+^ and CD8^+^ T cells between HIV-1-infected patients and HC are not significant different (*P* > 0.05, Table 1).

### Syphilis Infection Has Differential Effects on the Frequencies of γδ T-Cell Subsets in Patients with AHI

**Figure 3:**
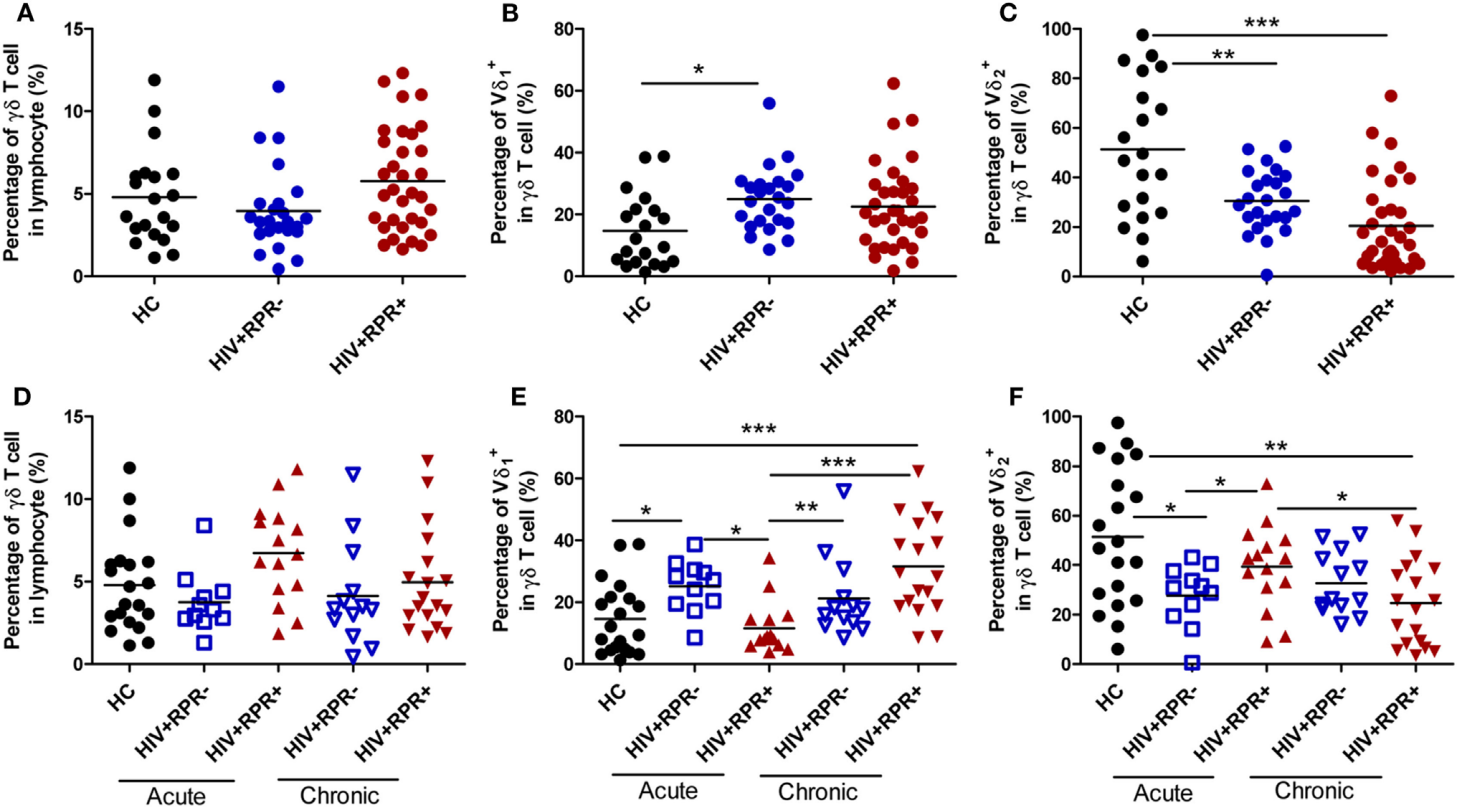
Comparison of the frequencies of γδ T cells, Vδ_1_ and Vδ_2_ T cells among healthy controls, HIV-1-infected and HIV-1/syphilis coinfected patients. Based on the results of rapid plasma reagin (RPR), HIV-1-infected patients were divided into HIV^+^RPR^−^ and HIV^+^RPR^+^ groups. The percentages of γδ T cells **(A)**, Vδ_1_ T cells **(B)**, and Vδ_2_ T cells **(C)** were compared among HC (●), HIV^+^RPR^−^ (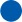), and HIV^+^RPR^+^ (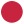) groups. Next, HIV^+^RPR^−^ and HIV^+^RPR^+^ groups were subdivided according to the acute or chronic nature of their HIV-1 infection. The percentages of γδ T cells **(D)**, Vδ_1_ T cells **(E)**, and Vδ_2_ T cells **(F)** were compared among HIV^+^RPR^−^ (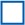) and HIV^+^RPR^+^ (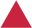) groups in acute HIV-1 infection and HIV^+^RPR^−^ (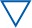) and HIV^+^RPR^+^ (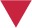) groups in chronic HIV-1 infection. The significance of differences was determined by calculating *P* values in Mann–Whitney tests and one-way ANOVA test. *P < 0.05, **P < 0.01, ***P < 0.001. HC, healthy controls; HIV^+^RPR^+^, patients coinfected with HIV-1 and syphilis; HIV^+^RPR^−^, patients infected with HIV-1 without syphilis.

First, we investigated the effect of syphilis on the frequencies of total γδ, Vδ_1_, and Vδ_2_ T-cell in HIV-1-infected patients. The frequencies of total γδ, Vδ_1_, and Vδ_2_ T cells among three groups: HC (*n* = 20), HIV^+^RPR^−^ (*n* = 24), and HIV^+^RPR^+^ (*n* = 33) patients were compared. As shown in Figure 3A, the frequencies of total γδ T cells were not significantly different among the three groups. The frequencies of Vδ_1_ T cells was significantly higher in the HIV^+^RPR^−^ group than in HC, but no significant difference was found compared with HIV^+^RPR^+^ group. The frequencies of Vδ_2_ T cells was significantly lower in both HIV^+^RPR^−^ and HIV^+^RPR^+^ groups compared with HC. However, we found no significant difference in Vδ_2_ T-cell frequency between HIV^+^RPR^−^ and HIV^+^RPR^+^ groups (Figures 3B,C). Based on the above results, we assumed that HIV-1 infection status might affect the frequencies of total γδ, Vδ_1_, and Vδ_2_ T cells. Therefore, we split the HIV^+^RPR^−^ and HIV^+^RPR^+^ groups into subgroups of patients with 26 AHI and 31 CHI patients. The frequencies of total γδ, Vδ_1_, and Vδ_2_ T cells were compared between the HIV^+^RPR^−^ and HIV^+^RPR^+^ subgroups with AHI and CHI. We found no significant difference of the frequencies of total γδ T cells between HIV^+^RPR^−^ and HIV^+^RPR^+^ groups for either acute or CHI (Figure 3D). The frequencies of Vδ_1_ T cells were significantly lower in the HIV^+^RPR^+^ group than in the HIV^+^RPR^−^ group for AHI patients. By contrast, in CHI, no significant difference of the frequencies of Vδ_1_ T cells was found between the HIV^+^RPR^+^ and HIV^+^RPR^−^ groups. Moreover, in the HIV^+^RPR^+^ group, the frequencies of Vδ_1_ T cells were significantly higher in CHI patients than in AHI patients (Figure 3E). Conversely, in AHI patients, the frequencies of Vδ_2_ T cells were significantly higher in the HIV^+^RPR^+^ group than in the HIV^+^RPR^−^ group. However, no significant difference of the frequencies of Vδ_2_ T cells between HIV^+^RPR^−^ and HIV^+^RPR^+^ groups was observed in CHI patients. Moreover, for HIV^+^RPR^+^ group, the frequencies of Vδ_2_ T cells in AHI patients were significantly higher than that in CHI patients (Figure 3F). Taken together, these results suggest that syphilis has differential effects on the frequencies of Vδ_1_ and Vδ_2_ T cells in patients with HIV-1 infection, depending on that this infection is acute or chronic.

### Syphilis Affects γδ T-Cell Differentiation in Patients with AHI

**Figure 4:**
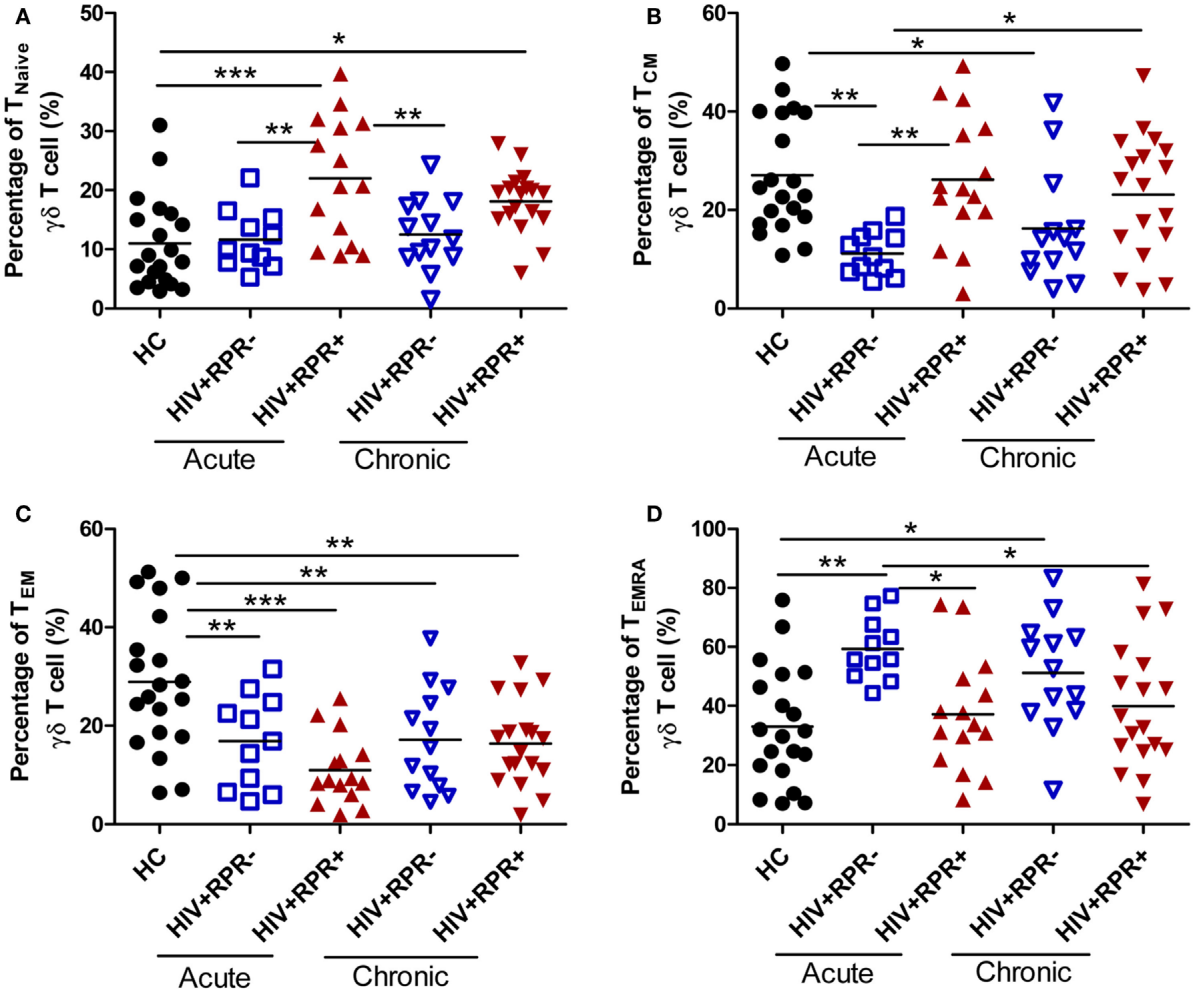
Syphilis coinfection has effects on the functional subsets of γδ T cells in HIV-1-infected patients. γδ T cells were classified into four different functional subsets according to their CD27 and CD45RA expression. The percentages of T_Naive_γδ T cells **(A)**, T_CM_ γδ T cells **(B)**, T_EM_ γδ T cells **(C)**, and T_EMRA_ γδ T cells **(D)** were compared among HC, HIV^+^RPR^−^ (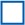), and HIV^+^RPR^+^ (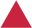) groups in acute HIV-1-infected patients and HIV^+^RPR^−^ (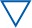) and HIV^+^RPR^+^ (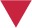) groups in chronic HIV-1-infected patients. The significance of differences was assessed by calculating *P* values in Mann–Whitney tests. *P < 0.05, **P < 0.01, ***P < 0.001. RPR, rapid plasma reagin.

Memory CD4^+^ T-cell depletion is a major cause of HIV-1 pathogenesis and disease progression (35, 36). Like CD4^+^ T cells, γδ T cells can be split into naïve and memory T-cell subsets on the basis of their surface expression of CD45RA and CD27 (37). Herein, we found that the frequencies of naïve (CD27^+^CD45RA^+^, T_Naïve_) γδ T cells in HIV^+^RPR^+^ group in AHI patients were significantly increased compared with HC, and HIV^+^RPR^−^ patients with both acute and CHI. The frequencies of T_Naïve_ γδ T cells in HIV^+^RPR^+^ group in CHI patients were significantly higher compared with HC, but no significant differences were shown compared with the other 3 groups (Figure 4A). In AHI patients, the frequencies of central memory (CD27^+^CD45RA^−^, T_CM_) γδ T cells in the HIV^+^RPR^−^ group were significantly lower than that in HC and HIV^+^RPR^+^ group. In CHI patients, the frequencies of T_CM_ γδ T cells were significantly higher than that in HIV^+^RPR^−^ group in AHI patients (Figure 4B). Moreover, the frequencies of effector memory (CD27^−^CD45RA^−^, T_EM_) γδ T cells in the HIV^+^RPR^−^ and HIV^+^RPR^+^ groups in both AHI and CHI patients were significantly lower than that in HC. However, there were no significant differences of the frequencies of T_EM_ γδ T cells between the HIV^+^RPR^−^ and HIV^+^RPR^+^ groups in both AHI and CHI patients (Figure 4C). Finally, we found the frequencies of terminally differentiated (CD27^−^CD45RA^+^, T_EMRA_) γδ T cells in HIV^+^RPR^−^ group in AHI patients were significantly higher than that in HC and HIV^+^RPR^+^ groups in both AHI and CHI patients. There was no significant difference between the HIV^+^RPR^−^ and HIV^+^RPR^+^ groups in CHI patients (Figure 4D).

Different γδ T-cell subsets exhibit different effector functions. CD27^−^ Vγ_9_Vδ_2_ T cells are considered as the major producer of IL-17 (38, 39) and neutrophils can regulate IL-17 production by γδ T cells (40–42). Therefore, we analyzed the relationships between the frequencies of T_Naïve_, T_CM_, T_EM_, and T_EMRA_ γδ T cells and the frequencies of neutrophils (Figure S1 in Supplementary Material). We found that the frequencies of T_EM_ γδ T cells were positively correlated with the frequencies of neutrophils. However, no relationships were observed between the frequencies of T_Naïve_, T_CM_, and T_EMRA_ γδ T cells and the frequencies of neutrophils (Figure S1 in Supplementary Material).

### Syphilis Coinfection Effects on γδ T-Cell Activation following Different Stages of HIV-1 Infection

**Figure 5:**
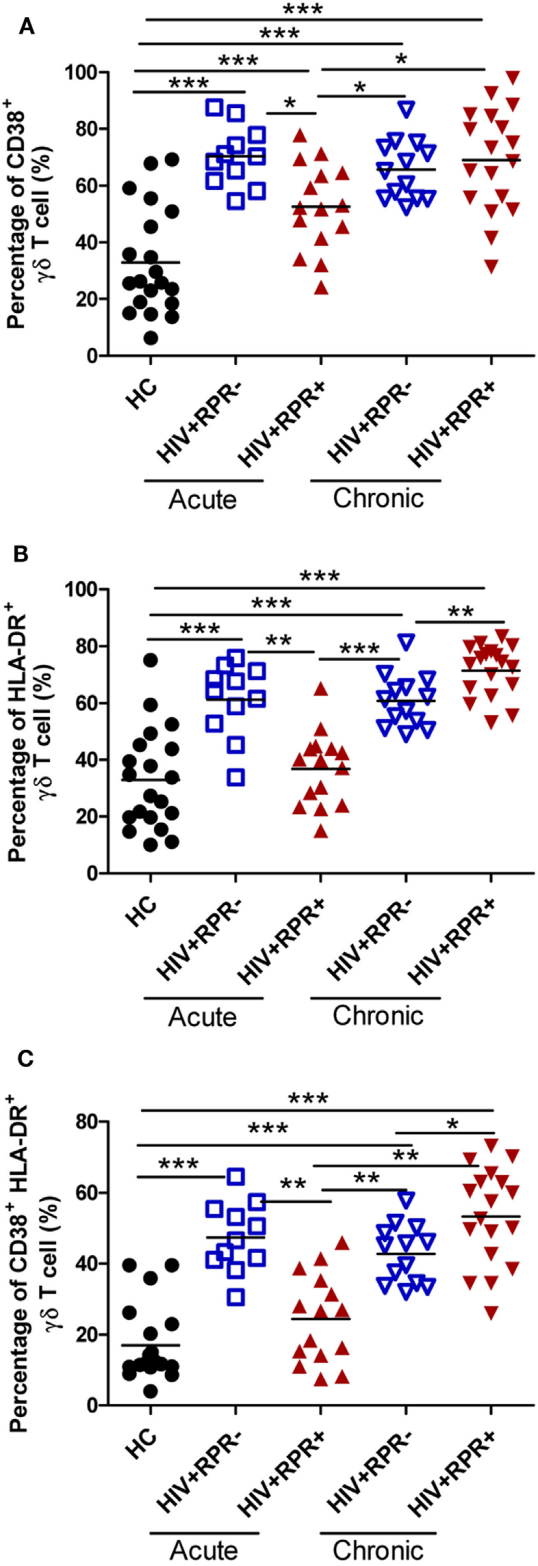
Syphilis coinfection affects differently γδ T-cell activation in patients with acute and chronic HIV-1 infection. γδ T-cell activation was assessed by evaluating the expression of CD38 and HLA-DR. The frequencies of CD38^+^ γδ T cells **(A)**, HLA-DR^+^ γδ T cells **(B)**, and CD38^+^HLA-DR^+^ γδ T cells **(C)** were compared among HC, HIV^+^RPR^−^ (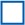), and HIV^+^RPR^+^ (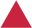) groups in acute HIV-1-infected patients and HIV^+^RPR^−^ (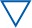) and HIV^+^RPR^+^ (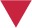) groups in chronic HIV-1-infected patients. The significance of differences was determined by calculating *P* values in Mann–Whitney tests. *P < 0.05, **P < 0.01, ***P < 0.001. RPR, rapid plasma reagin.

We have shown that γδ T cells were over-activated in HIV-1-infected patients, and associated with disease progression (26, 43). Here, we investigated the effects of syphilis on γδ T-cell activation by comparing γδ T-cell activation among HC, the HIV^+^RPR^+^ and HIV^+^RPR^−^ groups in both AHI and CHI patients. Consistent with our previous results, we found that the frequencies of CD38^+^γδ T cells were significantly higher in both the HIV^+^RPR^+^ and HIV^+^RPR^−^ groups in AHI and CHI patients, compared with HC. In AHI patients, the frequencies of CD38^+^γδ T cells in HIV^+^RPR^+^ group were significantly lower than that in HIV^+^RPR^−^ group (Figure 5A). In addition, we found that, in AHI patients, the frequencies of HLA-DR^+^ γδ T cells and CD38^+^HLA-DR^+^γδ T cells were significantly lower in the HIV^+^RPR^+^ group than in the HIV^+^RPR^−^ group (Figures 5B,C). By contrast, in CHI patients, the frequencies of HLA-DR^+^ γδ T cells and CD38^+^HLA-DR^+^γδ T cells were significantly higher in the HIV^+^RPR^+^ group than that in the HIV^+^RPR^−^ group (Figures 5B,C). Moreover, the frequencies of CD38^+^, HLA-DR^+^, and CD38^+^HLA-DR^+^γδ T cells in CHI patients, both HIV^+^RPR^+^ and HIV^+^RPR^−^ groups, were significantly higher than that in HIV^+^RPR^+^ group in AHI patients (Figures 5A–C). In a previous study, it was reported that neutrophils inhibited γδ T-cell activation (44), we therefore analyzed the relationship between γδ T-cell activation and the percentage of neutrophils. We found that there were no correlations between the frequencies of CD38^+^, HLA-DR^+^, and CD38^+^HLA-DR^+^ γδ T cells and the percentages of neutrophils (Figure S2 in Supplementary Material). Thus, HIV-1 infection appears to lead to γδ T-cell over-activation. However, syphilis can differentially affect γδ T-cell activation status, depending on the acute or chronic nature of the HIV-1 infection.

### IL-17-Producing γδ T Cells Are Positively Correlated with the Percentage of Neutrophils in HIV-1-Infected Patients

**Figure 6:**
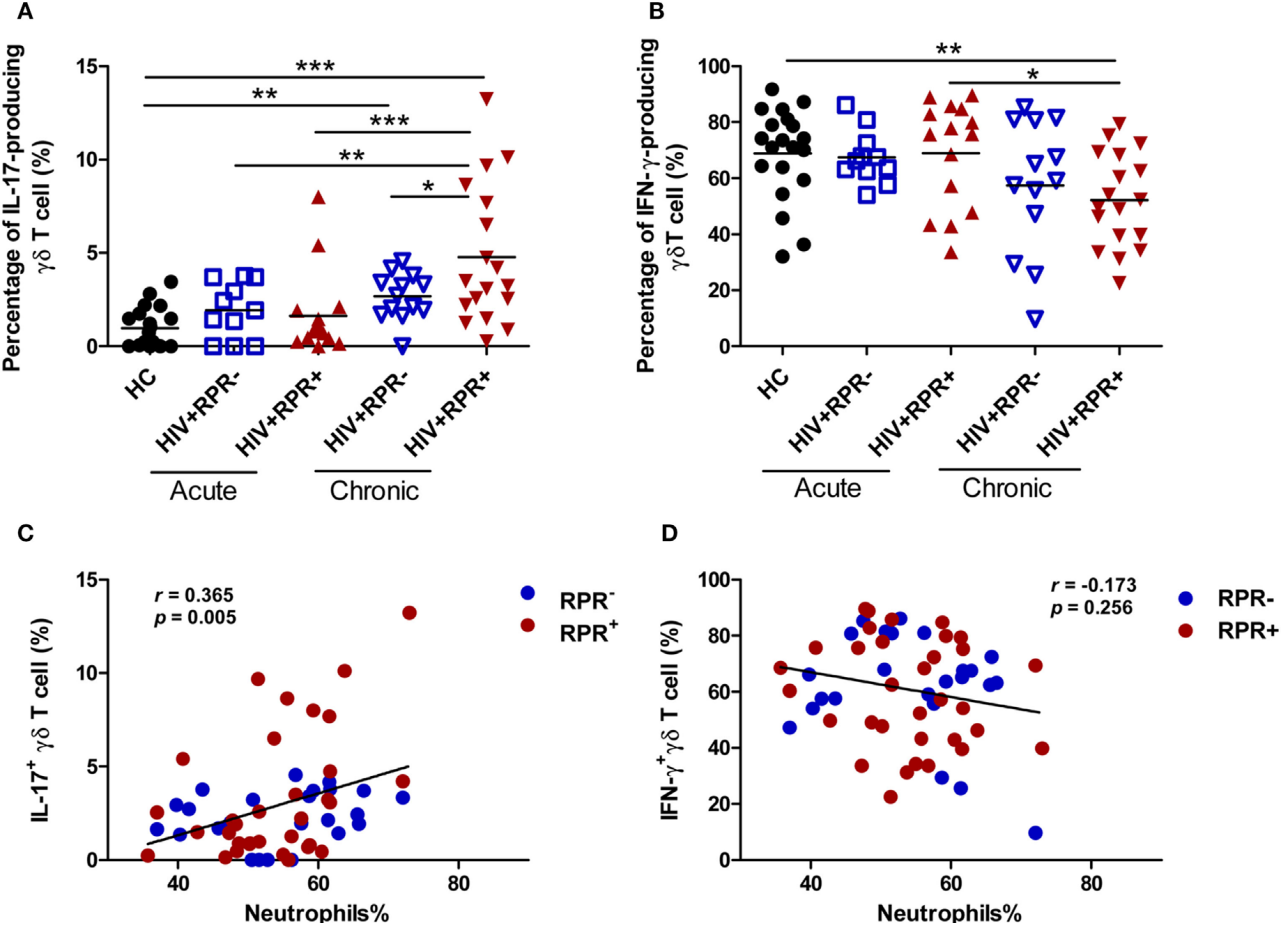
Syphilis coinfection promotes the production of IL-17 by γδ T cells in patients with chronic HIV-1 infection. Peripheral blood mononuclear cells (1 × 10^6^ cells/ml) were used to seed 24-well plates, and they were incubated with PMA (50 ng/ml)/ionomycin (1 µg/ml) for 6 h and BFA (10 µg/ml) was added 2 h before cell harvests. Intracellular staining for IL-17 and IFN-γ was assessed by flow cytometry. Comparisons of the frequencies of IL-17-producing γδ T cells **(A)** and IFN-γ-producing γδ T cells **(B)** among HIV^+^RPR^−^ (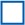) and HIV^+^RPR^+^ (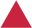) groups in acute HIV-1-infected patients and HIV^+^RPR^−^ (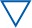) and HIV^+^RPR^+^ (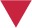) groups in chronic HIV-1-infected patients. Correlations of the proportions of IL-17-producing γδ T cells **(C)** and IFN-γ-producing γδ T cells **(D)** with the percentage of neutrophils in HIV^+^RPR^+^ (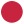) and HIV^+^RPR^−^ (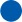) groups were analyzed. The significance of differences was assessed by calculating *P* values in Mann–Whitney tests. Spearman’s rank correlation analysis was used to assess correlations. *P < 0.05, **P < 0.01, ***P < 0.001. RPR, rapid plasma reagin.

Previous studies have shown that IL-17 levels in the peripheral blood or central nervous system (CFS) increase in patients with asymptomatic neurosyphilis and secondary syphilis, possibly as part of the immune response to *T. palladium* infection (19, 20, 27, 45). γδ T cells are a major source of IL-17, which is involved in inflammation and immune response (46, 47). Therefore, we assessed and compared the frequencies of IL-17-producing and IFN-γ-producing γδ T cells in HC, and in the HIV^+^RPR^+^ and HIV^+^RPR^−^ patients with acute and CHI. We found that the frequencies of IL-17-producing γδ T cells were significantly higher in CHI patients than in HC, particularly for the HIV^+^RPR^+^ group (Figure 6A). Surprisingly, we found a significant difference in the frequencies of IL-17-producing γδ T cells between the HIV^+^RPR^+^ and HIV^+^RPR^−^ groups was observed in CHI patients, but not in AHI patients. Moreover, the frequencies of IL-17-producing γδ T cells in HIV^+^RPR^+^ group in CHI patients were significantly higher than that in both HIV^+^RPR^+^ and HIV^+^RPR^−^ groups in AHI patients (Figure 6A). In addition, the frequency of IFN-γ-producing γδ T cells was significantly lower in the HIV^+^RPR^+^ group in CHI patients than that in HC. The frequencies of IFN-γ-producing γδ T cells in HIV^+^RPR^+^ group in AHI patients were significantly higher than that in HIV^+^RPR^+^ group in CHI patients. However, there was no significant difference in the frequency of IFN-γ-producing γδ T cells between the HIV^+^RPR^+^ and HIV^+^RPR^−^ groups in both AHI and CHI patients (Figure 6B). Thus, both IL-17 and IFN-γ may be involved in γδ T-cell-mediated immune response to syphilis, which seems to depend on HIV-1 disease stage. Recent studies have shown that neutrophils can suppress γδ T-cell activation, proliferation, and IFN-γ production (44, 48). However, Coffelt et al. reported that neutrophils promoted IL-17 production by γδ T cells, leading to tumor metastasis (49). We therefore hypothesized that the differences in γδ T-cell functions in AHI and CHI patients might be associated with neutrophils. We analyzed the relationships between IL-17- or IFN-γ-producing γδ T cells and the percentage of neutrophils. We found that the frequencies of IL-17-producing γδ T cells were positively correlated with the percentage of neutrophils (Figure 6C). However, no correlation was found between the frequencies of IFN-γ-producing γδ T cells and the percentage of neutrophils (Figure 6D). Taken together, these results suggest that syphilis may lead to the recruitment of neutrophils to local sites, where they promote the production of IL-17 by γδ T cells, leading to inflammation, immune activation, and an acceleration of HIV-1 disease progression.

## Discussion

HIV-1 and syphilis, two sexually transmitted diseases, show rapidly increasing incidences of coinfection in MSM population (2, 11). γδ T cells, a critical component of the host immune system, play an important role in controlling HIV-1 infection. However, the effects of syphilis on γδ T-cell phenotype and function remain unclear. Herein, we examined and compared the phenotype and function of γδ T cells in the HIV^+^RPR^+^ and HIV^+^RPR^−^ groups of patients with AHI and CHI separately. Consistent with previous studies (26, 50), we found that HIV-1 infection disrupted the balance of Vδ_1_ and Vδ_2_ T cells, increased the frequency of Vδ_1_ T cells, and decreased the frequency of Vδ_2_ T cells (Figures 3B,C). Interestingly, we found that syphilis corrected the imbalance of Vδ_1_ and Vδ_2_ T cells in patients with AHI, by decreasing the proportion of Vδ_1_ T cells and increasing that of Vδ_2_ T cells (Figures 3E,F). However, this effect was not observed in patients with CHI. The mechanisms by which syphilis affects the proportions of Vδ_1_ and Vδ_2_ T cells in patients with AHI are unknown, but may involve an induction of Vδ_1_ T-cell proliferation by *T. pallidum* antigens early in infection (51). In this study, we found that syphilis coinfection among HIV-1-infected patients did not affect the frequency of total γδ T cells; this may due to the mutual compensation of Vδ_1_ and Vδ_2_ T cells. The frequencies of Vδ_2_ T cells in healthy controls are diffusion, which appeared to be donor dependent. Vδ_1_ and Vδ_2_ T cells exhibit different features in location site, cytokine secretion, and cytotoxicity (24, 46, 52, 53). Therefore, it would be more helpful to understand the mechanisms of how syphilis differentially regulates the phenotype and function of γδ T cells by analyzing the phenotypes and functions of Vδ_1_ and Vδ_2_ T cells separately in the further studies.

γδ T cells can be classified into T_Naïve_, T_CM_, T_EM_, and T_EMRA_ four functional subsets. T_Naïve_ and T_CM_ γδ T cells are located in lymph nodes and short of immediate effector function, whereras T_EM_ and T_EMRA_ γδ T cells prefer to locate in inflammatory sites and show immediate effector functions (54). We found that the frequencies of T_Naïve_ and T_CM_ γδ T cells were significantly higher, but the frequencies of T_EM_ and T_EMRA_ γδ T cells were significantly lower in HIV^+^RPR^+^ group than those in HIV^+^RPR^−^ group in patients with acute HIV-1-infected patients (Figure 4). These results suggest that *T. pallidum* may trigger γδ T-cell proliferation and differentiation, in a manner dependent on the HIV-1 stage in coinfected patients, and that the changes of the frequencies of T_Naïve_, T_CM_, T_EM_, and T_EMRA_ γδ T cells may be associated with γδ T-cell activation (26). Furthermore, we observed a positive correlation between the frequencies of T_EM_ γδ T cells and the percentage of neutrophils in AHI. This may indicate that mutual interactions between immune cells may also have some effects on γδ T-cell differentiation.

Chronic immune activation is a key hallmark of HIV-1 infection and pathogenesis (55). We found here that γδ T cells are activated in all patients with both AHI and CHI, regardless of syphilis (Figure 5). However, syphilis infection regulates γδ T-cell activation status following the different stage of HIV-1 infection, that is, the frequencies of CD38^+^, HLA-DR^+^, and CD38^+^HLA-DR^+^ γδ T cells were decreased in patients with AHI but they were increased in patients with CHI (Figure 5). These differential effects may be mediated by the spirochetal lipoproteins, abundant membrane proteins inducing a strong immune response. Moreover, these lipoproteins, which enable the spirochetes to adhere to the host, determine the interaction between the spirochetes and the host environment and immune system (56). It has been suggested that spirochetal membrane lipoproteins bear pathogen-associated molecular patterns that bind to pattern recognition receptors such as Toll-like receptors (TLRs). Spirochetal lipoproteins (*T. pallidum* lipoproteins) are therefore pro-inflammatory. They may activate monocytes by binding to CD14, or macrophages through a TLR-dependent pathway, leading to the production of pro-inflammatory cytokines (57–60), which drive inflammation and induce γδ T-cell activation in patients with syphilis. The fact that syphilis differentially modulates γδ T-cell activation may be ascribed to the different host immune environments exhibited in patients with AHI and CHI. Moreover, interactions among immune cells may affect γδ T-cell activation (61). However, we did not observe any correlation between γδ T-cell activation and neutrophils in AHI. The precise mechanisms by which syphilis differentially modulates γδ T-cell activation are not fully understood and deserve further investigation.

IL-17, an inflammatory cytokine, has two principal effects: host protection and immunopathogenesis, with high levels leading to cancer progression and autoimmune diseases (62). Plasma IL-17 concentration increases in secondary syphilis, possibly as part of the immune response (20, 45). Syphilis also affects the liver, where it may cause syphilitic hepatitis or hepatic inflammatory tumors in HIV-1-positive MSM (63). However, the role of IL-17 and IL-17-producing γδ T cells in HIV-1-infected patients with syphilis remains unclear. In this study, we found that patients with CHI, particularly for the HIV^+^RPR^+^ group have an increase in the proportion of IL-17-producing γδ T cells, which was positively correlated with the percentage of neutrophils (Figures 6A,C). Our findings suggest that the immune response to *T. pallidum* promotes the production of IL-17 by γδ T cells in patients with CHI, leading to the recruitment of neutrophils to the inflammatory site, and to mediate the immune response (44, 63, 64). We previously showed that the frequency of IL-17-producing γδ T cells was correlated with γδ T-cell activation (26). We also show here that change of activated γδ T cells has a similar trend with the frequency of IL-17-producing γδ T cells in all patients (Figures 5 and 6). Together, these findings suggest that syphilis induces the production of IL-17 by γδ T cells, thereby promoting neutrophil-mediated immunity, triggering γδ T-cell immune activation, and accelerating HIV-1 disease progression. Neutrophils have recently been recognized as a source of IL-17 (40). However, the effects of neutrophils on γδ T cells remain unclear. Further studies on the γδ T-cell/IL-17/neutrophil axis are therefore required to elucidate the mechanisms of γδ T-cell action in HIV-1 pathogenesis and HIV-1 disease progression.

In summary, syphilis infection decreases immune activation and the secretion of inflammatory cytokines in AHI. To our knowledge, it’s the first time to reveal the role of γδ T cells in HIV-1 and syphilis coinfection. However, it remains unclear why and how syphilis differentially regulates the γδ T-cell immune response at different stages of HIV-1 infection. The different effects of syphilis on γδ T cells may relate to the different host immune environments in AHI and CHI and the natural features of γδ T cells which respond in the early stages of infection. Moreover, younger age other than CD4^+^ T-cell, nadir CD4^+^ T-cell, or CD8^+^ T-cell counts may be one of the key factors that affect the phenotype and function of γδ T cells (65). Therefore, syphilis should be followed up, monitored, and treated in the MSM population and in patients with AHI. In addition, there are still some limitations in this study. First, the sample size is small that may cause experimental deviation; second, we did not take into consideration other pathogens in this study, such as human cytomegalovirus and *Candida albicans* infection, which also have some effects on γδ T cells (66–69), although the medians of CD4 T-cell count among HIV-1-infected patients more than 400 cells/μl (Table 1). Therefore, larger samples and more detailed studies in further investigation are required to address these issues in depth. Our observations provide new insight into the roles of γδ T cells in immunopathogenesis of syphilis and HIV coinfection, particularly during AHI. Our findings may be helpful for the prevention of syphilis and other sexually transmitted infections, which is just the dedication in this research and highlights the great significance on the remedy of patients coinfected with HIV.

## Ethics Statement

This study and all relevant experiments have been approved by the Beijing You’an Hospital Research Ethics Committee and written informed consent was obtained from each participant according to the declaration of Helsinki. All the participants provided written informed consent for their information, and clinical samples were stored and used for research. At enrollment, all subjects provided baseline demographic, clinical and epidemiological information by completing a standardized questionnaire. The methods were carried out in accordance with approved guidelines and regulations.

## Author Contributions

ZL, HW, BS, and TZ conceived and designed the experiments; QZ, AS, YG, JY, and XH collected the sample information, contributed to reagents and materials; ZL, XL, ZH, ZWL, AS and DM performed the experiments and analyzed the data; and ZL, BS, WJ, HW, and TZ wrote the manuscript. All authors read and approved the final manuscript.

## Conflict of Interest Statement

The authors declare that the research was conducted in the absence of any commercial or financial relationships that could be construed as a potential conflict of interest.

## Supplementary Material

Figure S1 Correlations between the frequencies of T_Naive_, T_CM_, T_EM_, and T_EMRA_γδ T cells and neutrophils. γδ T cells can be divided into four functional subsets according to the expression of CD27 and CD45RA. There were no correlations between the frequencies of T_Naive_ **(A)**, T_CM_ **(B)**, T_EM_ **(C)**, and T_EMRA_ **(D)** γδ T cells and the percentage of neutrophils in all HIV-1-infected patients with RPR^−^ and RPR^+^. The frequencies of T_EM_ γδ T cells were positively correlated with the percentage of neutrophils in acute and all HIV-1-infected patients with RPR^−^ () and RPR^+^ (). Correlations were calculated by using Spearman’s rank correlation. *P*<0.05 was considered to be statistically significant. RPR, rapid plasma reagin.

Figure S2 Correlation between γδ T-cell activation and neutrophils. Two immune activation markers CD38 and HLA-DR were used to evaluate and compare γδ T-cell activation status in both HC and HIV-1-infected patients with RPR^−^ and RPR^+^. The relationship between γδ T-cell activation and neutrophils was analyzed. There were no correlations between the frequencies of CD38^+^ **(A)**, HLA-DR^+^ **(B)**, and CD38^+^HLA-DR^+^ **(C)** γδ T cells and the percentage of neutrophils in all HIV-1-infected patients with RPR^−^ () and RPR^+^ (). Correlations were calculated by using Spearman’s rank correlation. *P*<0.05 was considered to be statistically significant. RPR, rapid plasma reagin.
