# Supplementary figures and images for: Syphilis Infection Differentially Regulates the Phenotype and Function of γδ T Cells in HIV-1-Infected Patients Depends on the HIV-1 Disease Stage
Source: Front Immunol. 2017 Aug 21;8:991. doi: 10.3389/fimmu.2017.00991 (PMC5566620; doi:10.3389/fimmu.2017.00991)

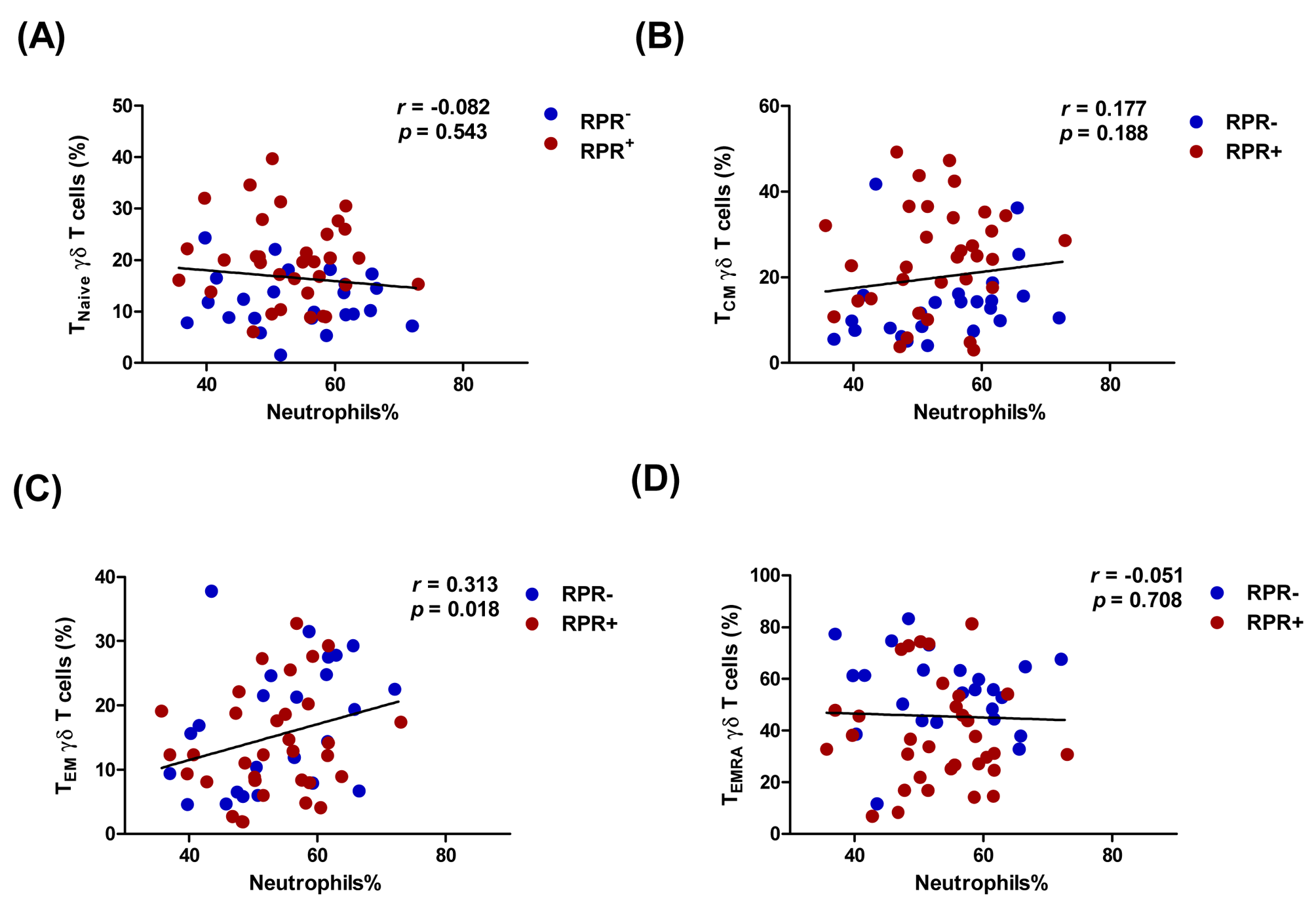

Supplement: Figure S1 — Correlations between the frequencies of TNaive, TCM, TEM, and TEMRAγδ T cells and neutrophils. γδ T cells can be divided into four functional subsets according to the expression of CD27 and CD45RA. There were no correlations between the frequencies of TNaive (A), TCM (B), TEM (C), and TEMRA (D) γδ T cells and the percentage of neutrophils in all HIV-1-infected patients with RPR− and RPR+. The frequencies of TEM γδ T cells were positively correlated with the percentage of neutrophils in acute and all HIV-1-infected patients with RPR− () and RPR+ (). Correlations were calculated by using Spearman’s rank correlation. P<0.05 was considered to be statistically significant. RPR, rapid plasma reagin. [file image_1.tif]

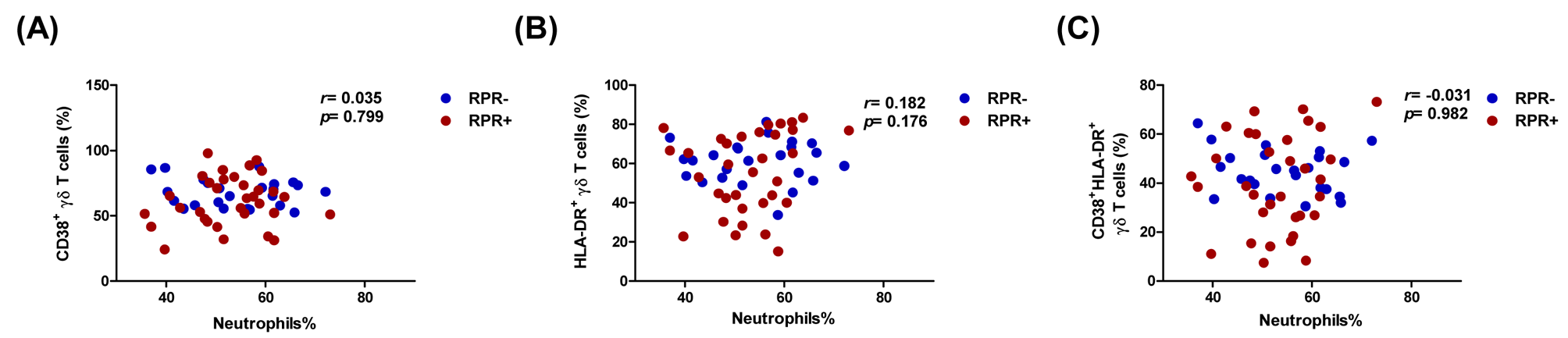

Supplement: Figure S2 — Correlation between γδ T-cell activation and neutrophils. Two immune activation markers CD38 and HLA-DR were used to evaluate and compare γδ T-cell activation status in both HC and HIV-1-infected patients with RPR− and RPR+. The relationship between γδ T-cell activation and neutrophils was analyzed. There were no correlations between the frequencies of CD38+ (A), HLA-DR+ (B), and CD38+HLA-DR+ (C) γδ T cells and the percentage of neutrophils in all HIV-1-infected patients with RPR− () and RPR+ (). Correlations were calculated by using Spearman’s rank correlation. P<0.05 was considered to be statistically significant. RPR, rapid plasma reagin. [file image_2.tif]
